# Medical Officers and Food Problems

**Published:** 1921-04-23

**Authors:** 


### Medical Officers and Food Problems.

A notable omission from the list of medical, scientific, labour, health, and philanthropic associations which recently joined in a memorial to the Ministry of Health on the subject of the diffusion of knowledge about diet and hygiene was the Society of Medical Officers of Health. We are glad to observe that this mistake has been acknowledged and that the Council of the Society has now decided to appoint a Committee to deal specially with food problems. In the current issue of the Journal of the Society an editorial article appeals to all persons interested in public health to support any movement which seeks to banish the appalling ignorance existing in all classes about foods. If anyone doubts the extnt of this ignorance he is invited to read the comments of the judge in the "Isle of Wight Official Case," where the judges decided that milk containing manure was "of the nature, substance, and quality" of the article demanded by the purchaser. "In other words, the purchaser is not considered by the learned judges to be prejudiced if he obtains manure in his milk."

We agree, and we have already expressed the

**THE PUBLIC WELL-BEING**—(*continued*).

opinion, that though voluntary organisations have done something to interest the public in these subjects, it is doubtful whether they can take the matter any further. It is very satisfactory to find that the Medical Officers' Society recognise the need for official intervention, and are prepared to lead the way. The medical officers of local authorities in this country have done splendid service in educating the public up to the importance of a pure-milk supply, and it lies specially within their power to effect a complete change in the public attitude towards the question of food in general. Any medical man who is familiar with the kind of meals eaten, for instance, in a typical Lancashire miner's family, cannot fail to realise how much human suffering and wastage occur solely through unsuitable diet. It is no exaggeration to say that if the women of the working classes possessed sound knowledge about food values a good deal of the hardships of under-employment and reduced wages and high prices would be mitigated, for it would be possible to spend shillings a week less on the family budget and at the same time considerably improve the nourishing quality of the food supplied to each member of the family.

In the same issue to which we have just referred attention is called to a matter which closely concerns the poorer elements of the community. This relates to the grading of milk, "which is naturally being accepted by the trade with complacency," though from a public point of view it has most serious defects, as it "merely adds another terror to poverty." The child of the rich, and the wealthy invalid, the writer points out, may be able, as the result of grading, to purchase milk absolutely above suspicion, but the poor will, unfortunately, be compelled always to buy the cheapest grade, which in this case will be particularly and peculiarly "cheap and nasty." If the Ministry of Health is to live and act up to its reputation, it will decide that the only way to protect the poor is to "make it an offence to sell milk that may reasonably be regarded as dangerous to health." The suggestion made is that the only satisfactory method from a public health point of view would be the general compulsory levelling-up of the standard of milk by such simple legislation as may be necessary in order to extend Section 6 of the Sale of Food and Drugs Act, so as to include bacterial and manurial contamination as an ingredient which is "to the prejudice of the purchaser."

